# Open Subpectoral Tenodesis for Isolated Traumatic Long Head of Biceps Tendon Rupture Provides Excellent Functional Outcomes in Active Male Patients

**DOI:** 10.7759/cureus.31553

**Published:** 2022-11-15

**Authors:** Christopher A Waugh, Tom Havenhand, Neil Jain

**Affiliations:** 1 Orthopaedics, Pennine Acute NHS Foundation Trust, Manchester, GBR; 2 Orthopaedics and Trauma, Pennine Acute NHS Foundation Trust, Manchester, GBR; 3 Trauma and Orthopaedics, Pennine Acute Hospitals NHS Trust, Manchester, GBR

**Keywords:** bicep pain, bicep tendon, tenodesis, biceps tenodesis, long head of biceps tendon

## Abstract

Background: For many years the long head of biceps tendon (LHBT) rupture has been described and is commonly identified by weakness, cramping, and the so-called "Popeye" sign. Traditionally, this was treated non-operatively, likely reflecting patient factors and the technical difficulty in reattaching a degenerative and shortened tendon. In contrast, traumatic distal biceps rupture is now commonly repaired despite historically being managed non-operatively. The advent of a convenient and reproducible surgical technique led to an increase in the rate of fixation, thereby improving the cramping and weakness associated with non-operative treatment. Given recent surgical advances within this field, many techniques are now present for LHBT pathology. We describe results from a cohort of patients suffering traumatic LHBT rupture who sought a surgical solution to improve their symptoms.

Methods: Over four years, 18 male patients underwent surgical intervention for isolated traumatic LHBT rupture. The technique used involved an open subpectoral tenodesis with fixation of the LHBT into the bicipital groove. Postoperative immobilization using a sling was recommended for six weeks prior to a progressive rehabilitation program. Patients were assessed with pre- and postoperative visual analog scores (VAS) for pain and American Shoulder and Elbow Society (ASES) scores.

Results: The mean patient age at the time of surgery was 49 years (range: 26-65 years). The mean time to surgery was nine weeks (range: 2-24 weeks). All patients showed an improvement following surgery with a mean pre-op ASES score of 33 (range: 10-60) compared to a post-op score of 92.6 (range: 85-100). All patients were able to return to work and sport, with all but one returning to the same functional demand level of work. The mean pre-op pain VAS was 6.3 (out of 10) compared to 0.2 post-op. All patients had a requirement for analgesia pre-operatively and none had postoperatively. No surgical complications were observed. No correlation was observed between the time to surgery and the outcome.

Discussion: LHBT rupture is often treated non-operatively as few studies within the literature describe the surgical technique and outcomes from surgical intervention. When treated non-operatively, patients complain of pain, cramping, and cosmetic deformity known as the "Popeye" sign. Following a traumatic rupture of the LHBT, we have demonstrated excellent outcomes using a standard approach and common fixation technique that has the potential to improve the functional outcome for symptomatic patients.

Conclusion: Open subpectoral biceps tenodesis is associated with excellent outcomes in symptomatic patients following isolated LHBT rupture.

## Introduction

Traumatic rupture of the long head of biceps tendon (LHBT) has been extensively described as a common cause of anterior shoulder pain and instability [1]. The proximal biceps brachii origin is composed of two tendinous heads: a short head originating from the coracoid process and a long head inserting at the supraglenoid tubercle and superior glenoid labrum [2]. The distal biceps tendon inserts on the bicipital tuberosity of the radius. The influence of biceps brachii on arm function remains disputed, though it likely contributes to composite movements including strong forearm supination, weak elbow flexion, and stabilization of the glenohumeral joint [3].

The majority of traumatic biceps brachii ruptures involve the LHBT [4]. Such injuries often occur with concomitant superior labral anterior-posterior (SLAP) lesions, rotator cuff tears, tendinitis, and tenosynovitis [5]. Patients often complain of pain, cramping, weakness, and visible deformity [5]. Clinical diagnosis is usually made based on a history of trauma and a pathognomonic "Popeye" sign on clinical examination. However, high-resolution ultrasound scan shows high specificity and is being increasingly utilized for diagnosis [1].

Traumatic LHBT rupture was historically treated non-operatively with conservative measures such as non-steroidal anti-inflammatory drugs (NSAIDs), corticosteroid injection, and physiotherapy [4]. This likely reflected an elderly patient population, the prevalence of rheumatological disease, and the technical challenge of inserting a short, degenerative tendon into the superior glenoid labrum. Patients were left with residual cosmetic deformity and often debilitating intermittent cramping. In contrast, distal biceps brachii tendon ruptures occur more often in younger patients and are generally managed surgically [6].

Recent advances in surgical technique have led to the development of convenient, reproducible fixation options for LHBT pathology. Tenotomy and tenodesis are two such options, though the effectiveness of one above the other remains disputed [7]. It has been proposed that tenodesis may lead to better patient outcomes by improving the shape, strength, and length-tension relationship of the pathological LHBT though this remains controversial [8]. Ultimately, the preference for either technique reflects a process of shared decision-making between the patient and surgeon.

Biceps tenodesis poses a number of important technical challenges to surgeons. For isolated LHBT ruptures, open tenodesis is the most commonly performed procedure [9]. There is some suggestion that the use of a suture anchor rather than interference screw fixation may lead to better outcomes due to greater biomechanical stability [10]. A subpectoral rather than suprapectoral tenodesis site appears to favor lower re-operation rates, with patients experiencing less pain, given that LHBT is released from the bicipital groove [11]. Though generally rare, complications can range from postoperative stiffness and swelling to rarer reports of humeral shaft fractures and brachial plexus injury [12-16].

Following traumatic rupture of the LHBT, we propose a common fixation technique using a standard approach that has the potential to help many patients. We hypothesize significantly improved postoperative outcomes for patients using a well-established assessment tool that has been validated for a variety of shoulder pathologies [13].

## Materials and methods

Over a four-year period (2016-2020), 18 male patients underwent surgical intervention for isolated traumatic LHBT rupture. Upon clinical assessment, all patients gave a history of chronic symptoms leading to a traumatic event that left them with significant pain and decreased functional ability in their arms. Each described a resultant new visible deformity, and a clinical exam revealed a "Popeye sign." Additionally, each patient had subjective weakness and pain on resisted forearm supination at 90 degrees of elbow flexion. Cases with concomitant shoulder joint pathology or instability were excluded from this study.

A diagnosis of traumatic rupture of the long head of the biceps tendon was confirmed in all patients using magnetic resonance imaging (MRI). The same sequence was performed for all patients including axial proton density (PD) fat saturation (FS), coronal T2 FS and PD, sagittal T2 FS, and PD sequences. All patients failed to show improvement in their symptoms with conservative treatment. A shared decision-making model was adopted for treatment, and these patients offered their appropriately informed consent to proceed with surgery in the form of an open subpectoral tenodesis, given their dissatisfaction with their current level of symptoms. All surgeries were performed by the senior author who is fellowship trained in sports trauma and soft tissue orthopedics and has over a decade of experience.

All patients were given strict guidance on postoperative immobilization, rehabilitation, and follow-up. Postoperative outcomes were assessed following discharge to the community.

Surgical technique

**Figure 1:**
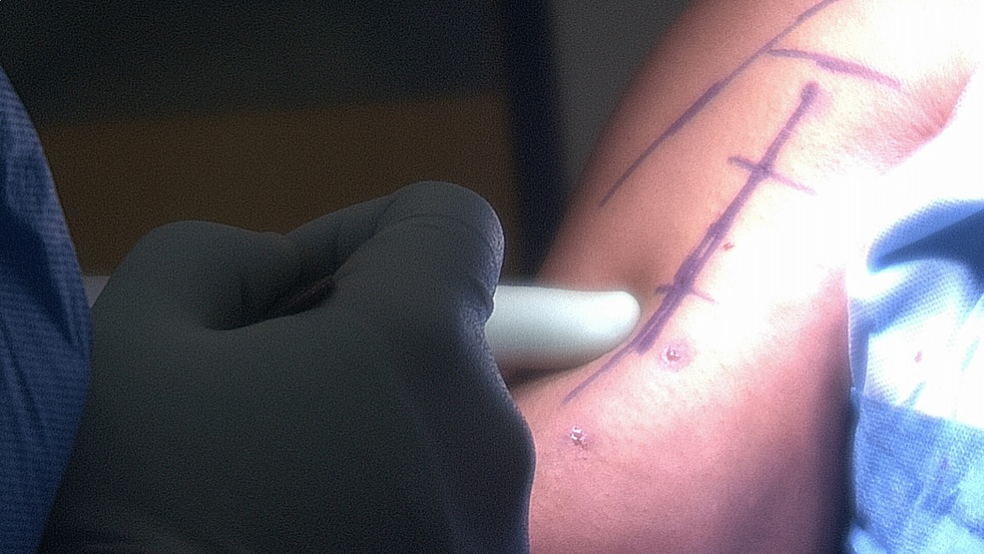
A proximal deltopectoral incision is performed just below the inferior border of pectoralis major, lateral to the axillary crease

The patient is placed in a beach-chair position under general anesthetic. An examination under anesthesia is used to assess the degree of passive movement and stability. The entire arm is prepped, draped, and positioned within a mechanical arm fixator in mild abduction and flexion. Local anesthetic with adrenaline is introduced along a marked incision line to aid hemostasis. A proximal deltopectoral incision is performed just below the inferior border of the pectoralis major, lateral to the axillary crease (Figure 1).

The soft tissues are dissected down to expose the distal bicipital groove. The deltoid is retracted laterally exposing the bicipital groove as the anatomical landmark to base the surgical exploration. It should be stated that the LHBT was found to be in variable positions at this point.

**Figure 2:**
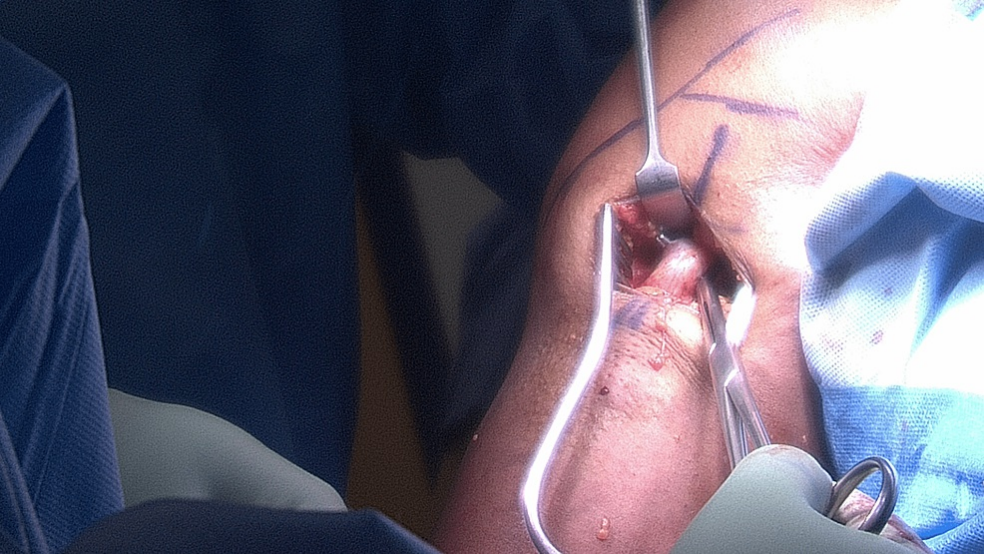
The long head of the biceps tendon remains within the bicipital groove, retracted to the point of the transverse humeral ligament

Our experience would term the following: (1) one of three common positions remains in the groove having detached from the glenoid but retracted to the point of the transverse humeral ligament (THL) (Figure 2). These cases were simple to free from the THL in the acute setting. When the surgery was performed more than six weeks after the injury, there was some fibrosis between the LHBT and THL that required liberating in order to mobilize the LHBT. (2) The LHBT is retracted to the mid-upper arm level and was found to be in a spiral position. Often, this was within a fibrotic covering of seroma if the case was performed within four weeks. This was straightforward to open and reveal a near-normal tendon and length within it. (3) The LHBT is retracted to mid-arm and found to be encased within a fibrotic ball. This was found in chronic cases of more than four weeks. There was a difficult dissection within the fibrotic ball that was required to liberate an LHBT within that appeared macroscopically to be shortened and of poorer quality than one would see in a more acute case.

**Figure 3:**
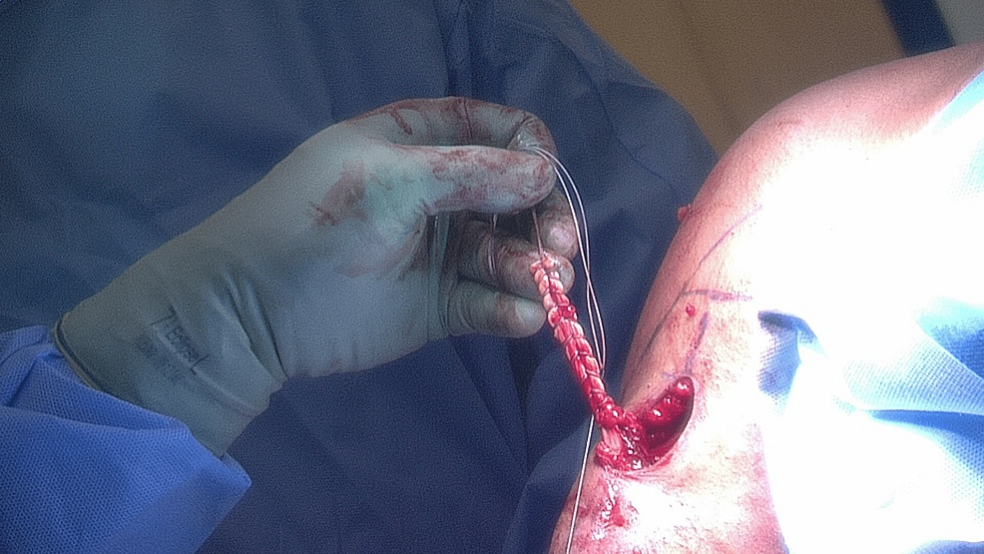
The long head of biceps tendon is sutured in a retrograde fashion from the musculotendinous junction proximally with a continuous loop suture

Once identified and liberated, the LHBT is sutured in a retrograde fashion from the musculotendinous junction proximally to the often frayed proximal end with a continuous loop suture (#2 FiberLoop; Arthrex, Naples, Florida) (Figure 3).

**Figure 4:**
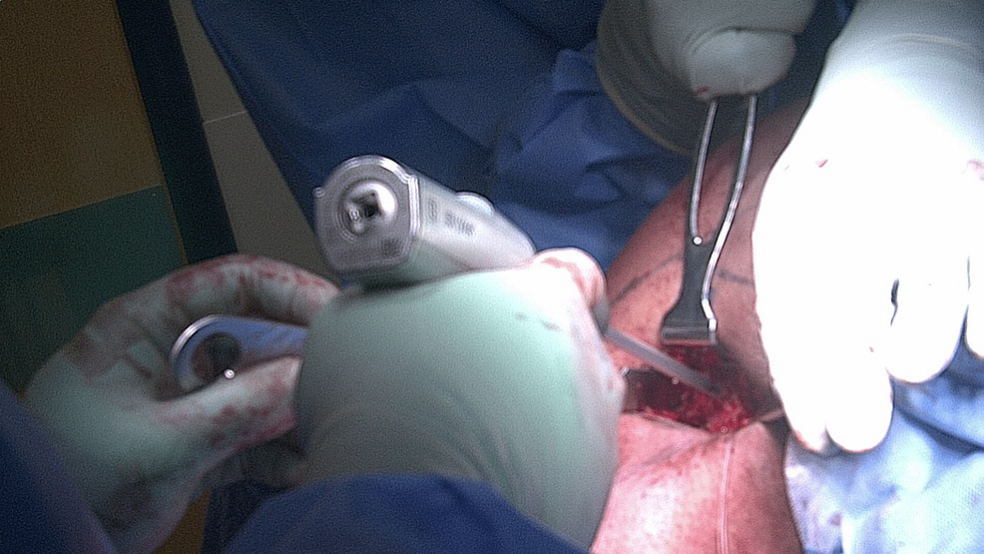
A unicortical socket is prepared using a pilot-tipped reamer

The tenodesis insertion site was then identified in the distal bicipital groove (DBG). A unicortical socket is prepared using a pilot-tipped reamer (Figure 4).

**Figure 5:**
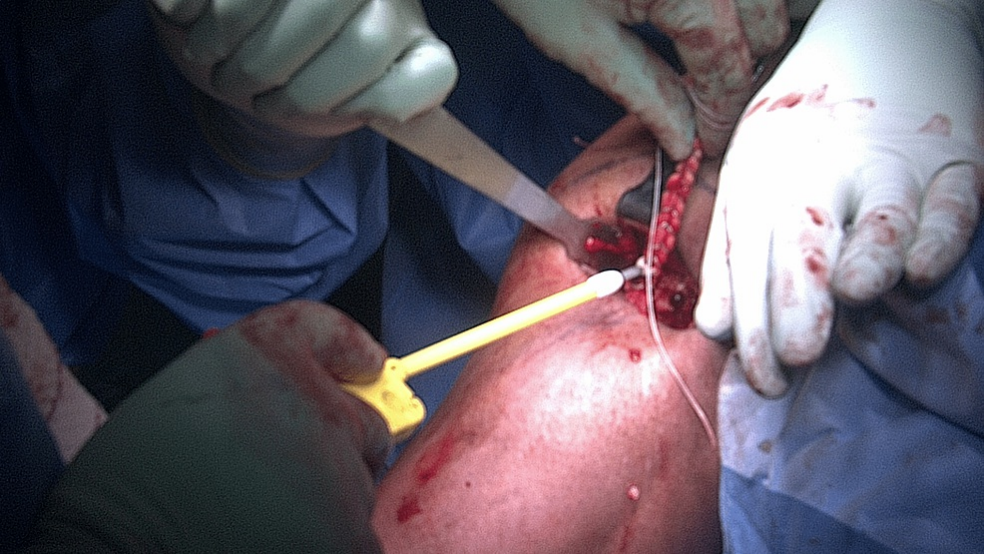
The anchor-tendon-suture construct is introduced into the bone socket as close to the musculotendinous junction as possible in order to achieve suitable tension in the biceps

An additional suture (#2-0 Fiberwire; Arthrex, Naples, Florida) is then looped through the eyelets of the fork in a biocomposite forked swivel-lock anchor (Arthrex, Naples, Florida), and the LHBT is placed within the loop. The anchor-tendon-suture construct is then introduced into the bone socket at a level as close to the musculotendinous junction as possible in order to achieve suitable tension in the biceps (Figure 5).

Any remaining proximal portion of the LHBT is excised. The wound is irrigated before layered closure using Ethibond and Vicryl sutures.

Postoperative rehabilitation and follow-up

Immobilization using a sling was recommended for six weeks, prior to a progressive rehabilitation program under the supervision of a physiotherapist. Initially, actively assisted flexion and extension exercises for the elbow were advised. At week 9, active elbow flexion and extension without resistance were advised. By week 12, patients were instructed to gradually increase resistance as pain allows and were prescribed a program to increase muscle activity using resistance bands. At week 16, the patients were permitted to return to their usual activity level if they felt able.

Patients were seen in the clinic for wound review and suture removal at two weeks post-procedure. Subsequent follow-ups occurred on the 2nd, 6th, and 12th months before discharge from service.

Data collection

Data were collected retrospectively for all patients who underwent surgical intervention. Each patient was asked to complete an American Shoulder and Elbow Society (ASES) score comparing their level of function six months prior to and one year following surgery. Consent was obtained prior to a short telephone interview with a practitioner independent of the surgeon performing the procedure. Answers provided were used to calculate a postoperative ASES score and University of California at Los Angeles Shoulder Rating Scale (UCLA) score. VAS for pain was also recorded. The pre- and postoperative scores were compared.

## Results

Patient demographics

The mean age of patients at the time of surgery was 49 years (range: 26-65). All patients were males and performed a variety of manual jobs prior to the injury. The average time to surgery from initial injury was nine weeks (range: 2-24). The average time to discharge was 26 months (range: 12-36 months). Two patients were lost to follow-up.

Peri-operative functional assessment

All patients reported significant improvements in pain and function postoperatively (p = <0.05). The mean pre-op ASES score was 33 (range: 10-60). The mean post-op ASES score was 92.6 (range: 85-100). All patients were able to return to work, with all but one returning to the same functional demand level (p = <0.05). All complained of difficulty with sport pre-operatively compared to one postoperatively (p = <0.05). All described difficulty in work pre-operatively in contrast with none postoperatively (p = <0.05). The mean pre-op pain VAS was 6.3 (out of 10) compared to 0.2 post-op (p = <0.05). All patients had a requirement for analgesia pre-operatively and none had postoperatively (p = <0.05). Six reported shoulder pain at night pre-operatively compared to three postoperatively, failing to reach significance (p = 0.31). The average number of analgesia pills taken per day pre-operatively was 5.2 compared to 1.2 pills postoperatively (p = <0.05). All patients reported difficulty putting on a coat pre-operatively compared to three postoperatively (p = 0.44). All patients reported significant improvement in their postoperative ability to perform tasks such as sleeping on the affected side, washing their back, combing their hair, and throwing a ball overhand (p = <0.05). No significant surgical complications or failures were observed. All patients reported improved cosmetic appearance at the time of discharge. No correlation was observed between the time to surgery and the outcome.

## Discussion

The objective of this study was to assess the utility of performing an open subpectoral tenodesis of the LHBT for active older patients with chronic traumatic rupture. This condition is often treated non-operatively in older patients as there are few studies that describe both the surgical technique and outcomes resulting from operative intervention [2]. When treated non-operatively, patients complain of debilitating pain and cramping in the biceps along with obvious cosmetic deformity. The most important finding is that all patients were able to return to greater activity levels and had less pain postoperatively as evidenced by their ASES and VAS scores.

LHBT tenotomy and tenodesis both demonstrate excellent outcomes, though neither technique has been proven superior. The majority of biceps tenodesis studies compare a range of fixation techniques, with a lack of power and randomization. Ng and Funk reported on 11 patients with chronic LHBT rupture who underwent tenodesis. In 60% of cases, they were unable to perform interference screw fixation due to insufficient tendon length, which we did not observe [17]. Tangari et al. demonstrated excellent outcomes following an acute rupture in five patients using a mini-open tenodesis with suture anchors [18]. Our study describes a novel fixation technique using a standard approach that has the potential to help active older male patients with chronic symptoms.

Arthroscopic biceps tenodesis has become an established treatment option for young, active patients with acute, traumatic LHBT pathology [19,20]. The chosen fixation technique is dependent upon position, mobility, and quality of the LHBT as well as patient and surgeon preference. However, previous studies have failed to demonstrate a significant difference in the outcomes between open and arthroscopic repair [21]. In older patients, there is a paucity of data regarding the outcomes for patients with chronic rupture. Within our cohort, the presence of a chronically degenerative and retracted LHBT precluded arthroscopic fixation.

Anterior humeral pain is a well-documented complication resulting from LHBT tenodesis [22]. Gregory et al. suggested that pain around the site of tenodesis may persist in more than half of the cases [23]. However, the novel surgical fixation technique demonstrated in this study shows significant improvement in VAS for pain and ASES scores. We did not observe any surgical complications of note. These patients reported that outcome measures have been used extensively to assess general shoulder function.

Our study population focused exclusively on patients with isolated chronic LHBT rupture, confirmed through MRI. We have demonstrated significant improvements in postoperative pain and functional capacity while mitigating the effects of concomitant shoulder pathology, following LHBT tenodesis. These effects were demonstrated over an average time to discharge of 26 months, with only two patients lost to follow-up (89%).

Our study is limited by a relatively small sample size, which reflects that many patients with LHBT rupture are managed without surgery. We acknowledge that our data were non-randomized, and we report on an older, active population that may reflect selection bias. All patients within our cohort were males, which may reflect selection bias due to higher physical demands or psychosocial factors associated with having a "Popeye sign." In addition, our case series did not include a control group for direct comparison. There is a potential for performance bias, given that all procedures were performed by a single surgeon. Finally, we did not assess other measures such as radiological (e.g., tendon position) or biomechanical (e.g., strength) outcomes.

Subsequent studies including larger patient cohorts with a broader age range and utilizing a randomized control trial framework would be useful in determining if this treatment method could be utilized in a wider selection of patients to improve the outcomes when compared to the current standard of care, which is predominantly non-operative.

## Conclusions

Open subpectoral LHBT tenodesis improves pain and function in older, active male patients following isolated chronic rupture. All patients were able to return to a higher level of physical demand as evidenced by their VAS and ASES scores for pain. Hence, we would recommend its use in active, older symptomatic patients.
